# Potential COVID-19 test fraud detection: Findings from a pilot study comparing conventional and statistical approaches

**DOI:** 10.25646/12100

**Published:** 2024-06-19

**Authors:** Michael Bosnjak, Stefan Dahm, Ronny Kuhnert, Dennis Weihrauch, Angelika Schaffrath Rosario, Julia Hurraß, Patrick Schmich, Lothar H. Wieler

**Affiliations:** 1 Trier University, Department for Psychological Research Methods, Trier, Germany; 2 Robert Koch Institute, Department of Epidemiology and Health Monitoring, Berlin, Germany; 3 City of Cologne, Health Authority, Infectious and Environmental Hygiene, Cologne, Germany; 4 Digital Global Public Health at the Hasso-Plattner-Institute (HPI), University of Potsdam, Potsdam, Germany

**Keywords:** COVID-19 Test Billing, Healthcare Billing Fraud, Statistical Fraud Detection

## Abstract

**Background:**

Some COVID-19 testing centres have reported manipulated test numbers for antigen tests/rapid tests. This study compares statistical approaches with traditional fraud detection methods. The extent of agreement between traditional and statistical methods was analysed, as well as the extent to which statistical approaches can identify additional cases of potential fraud.

**Methods:**

Outlier detection marking a high number of tests, modeling of the positivity rate (Poisson Regression), deviation from distributional assumptions regarding the first digit (Benford’s Law) and the last digit of the number of reported tests. The basis of the analyses were billing data (April 2021 to August 2022) from 907 testing centres in a German city.

**Results:**

The positive agreement between the conventional and statistical approaches (‘sensitivity’) was between 8.6% and 24.7%, the negative agreement (‘specificity’) was between 91.3% and 94.6%. The proportion of potentially fraudulent testing centres additionally identified by statistical approaches was between 7.0% and 8.7%. The combination of at least two statistical methods resulted in an optimal detection rate of test centres with previously undetected initial suspicion.

**Conclusions:**

The statistical approaches were more effective and systematic in identifying potentially fraudulent testing centres than the conventional methods. Testing centres should be urged to map paradata (e.g. timestamps of testing) in future pandemics.

## 1. Introduction

### 1.1 Background

The COVID-19 pandemic has highlighted the critical importance of rapid and extensive antigen testing in managing the spread of the virus [1].

The demand for extensive COVID-19 testing capacities was met in almost all countries by offering financial incentives to existing and newly established test centres. For instance, in Germany, a legal regulation (‘Corona Test Regulation’, TestV [2]) guaranteed reimbursement of testing from tax funds. While such incentives – possibly in combination with insufficient fraud prevention measures – have decisively contributed to building up extensive testing capacities rapidly, it has also opened up a pandora’s box in terms of fraudulent activities, causing financial damage to tax payers. Specifically, it has been reported in public media that some testing centres overreported the number of actually conducted COVID-19 tests, and/or invoiced test appointments that were scheduled, but then canceled [3]. It has also been reported that some fraudulent testing centres did not even exist (no actual place of business), but still managed to get their fraudulent invoices reimbursed [4]. Given such media reports, it was decided by the Federal Ministry of Health to establish various measures and procedures to identify potentially fraudulent COVID-19 test centres, among them a set of statistical approaches identifying suspicious test centres.

The overall aim of this paper is to report on a pilot study conducted at the Robert Koch Institute comparing the performance of different statistical approaches to identify potentially fraudulent test centres in relation to conventional fraud detection procedures used by local health authorities by default, using data from one German health authority in a city with more than 900 test centres. This pilot study may serve as a basis for the widespread application of statistical approaches to uncover potentially fraudulent testing centres, and at the same time serves as a contribution to a more accurate representation of testing activities in future pandemics.

### 1.2 Research question

There are around 400 local health authorities in Germany. In the event of an outbreak, the local health authorities play a key role and decide on and implement appropriate measures. During the COVID-19 pandemic, they also had the task of monitoring test centres and forwarding information on potentially fraudulent activities to the law enforcement authorities. Test centres were usually suspected of fraud by health authorities if they met at least one of the following criteria: excessively large test numbers, significant deviation of the proportion of positive test results (positive rate) from the average, or complaints by whistleblowers (citizens, current or past employees from the test centres). In addition, test centres were also considered suspicious of fraud when they were the subject of police or prosecutorial investigations. Typical occasions for such investigations were reports by citizens and whistleblowers. Banks that recorded unusually high movements in test centre bank accounts also served as whistleblowers for the initiation of investigations.

These traditional approaches are time-consuming, hardly uniformly implemented, and resource-intensive. The question arises whether potentially fraudulent COVID-19 testing centres can also be identified based on billing data using systematically applied statistical approaches, and thus contribute to the efficient use of available resources. Accordingly, we pursued two research questions in our pilot study: First, what is the level of agreement between conventional methods of identifying suspected test centre fraud and a set of statistical methods applied to data on claims for COVID-19 antigen tests? Second, for a set of test centres identified as suspicious by our statistical approaches, what is the proportion of those for which there had been no suspicion of fraud by conventional methods and for which the suspicion raised by statistical methods could be confirmed after a thorough criminal investigation?

## 2. Methods

### 2.1 Data

We used data on claims for COVID-19 antigen tests submitted for reimbursement by 907 test centres operating in a German city with approximately one million residents for the timespan April 8, 2021 through August 28, 2022. The data were transmitted on a daily basis via an online portal provided for this purpose by the ministry of a federal German state. Transmission was mandatory by law for the test centres. For each claim, the following information was provided: test centre category (pharmacy, doctor’s or dentist’s office, private test centre), date of testing, number of tests performed per day, number of positive tests per day. Table 1 summarises the frequencies and descriptives for the data used in this pilot study.

### 2.2 Conventional versus statistical methods

#### Conventional method: Suspicion of fraud by the health authorities

Test centres suspected of fraud were classified according to the following indicators: Due to apparently excessively large test numbers, significantly low positive rates, or whistleblowers (citizen complaints, current or past employees from the test centres), on-site inspections were initiated that could not dispel the suspicion of fraud. In addition, test centres were also considered to be suspected of fraud when they are the subject of police or prosecutorial investigations. All these conventional procedures were implemented sporadically and lack a systematic approach. The scope and intensity of the audits were heavily reliant on the existing human and financial resources.

#### Statistical conspicuousness of fraud

A test centre was classified as statistically conspicuous of fraud based on at least one of the following four indicators used: High number of tests invoiced by a specific test centre compared to the mean number of tests per day (stratified by test centre category: pharmacies, doctor’s or dentist’s offices, private test centres), low positive rate of a test centre within a test centre category (analysed with a Poisson regression), deviations of reported data from Benford’s law or from the assumption of equally distributed last digits. This new set of statistical approaches, as compared to the conventional one employed, represents a systematic workflow consisting of a series of statistical test procedures applied to all test centres. Each statistical method is further described below.

### 2.3 Outcomes

Three outcome variables have been computed in this study. First, the percentage of positive overlap (formula 1) between the conventional and statistical methods (paralleling the concept of sensitivity in test accuracy studies [5]):


(1)

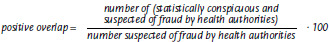



The second outcome used is the percentage of negative overlap (formula 2) (paralleling the concept of specificity in test accuracy studies [5]):


(2)

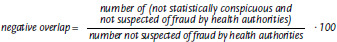



Third, the share of incrementally identified potentially fraudulent test centres by the new method, over and above those identified by conventional approaches (formula 3) (corresponding to 1 minus specificity resp. 100 minus negative overlap):


(3)

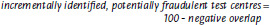



### 2.4 Statistical methods to identify potential fraud

The following statistical approaches have been applied to the data. A more comprehensive description can be found in [6].

#### Outlier identification based on a high number of tests invoiced

**Figure 1:**
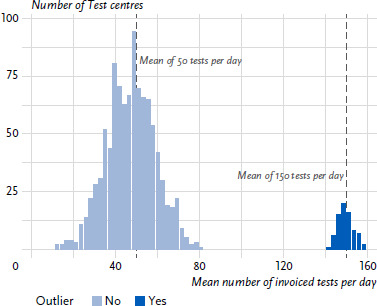
Histogram of two simulated distributions: Mean number of invoiced tests with outlier identification. Source: Own figure

Test centres with an unusually high average number of invoiced tests per day compared to the other test centres within the same category (pharmacy, doctor’s or dentist’s office, private test centre) were classified as conspicuous. The limit was set at the 90% percentile depending on the test centre category. The histogram in Figure 1 visualises outliers, i.e. suspicious test centres (marked dark blue).

#### Poisson regression for outlier identification through suspiciously low positive rates

This approach determines the extent to which individual testing centres report a positive rate that is too low. The suitability of the low positive rate of the testing centres as an indicator of fraud can be attributed to the fact that a positive test resulted in conditions being imposed on the person concerned. A negative test would therefore have encouraged fraud by not leading to further action.

The number of positive tests per day and test centre was modeled by a Poisson regression with random effects. Poisson regression is a type of regression analysis used to model count data [7, 8]. It can also be used to model the positive rate, i.e. the proportion of positive tests per day and test centre, by including the total number of tests per day and test centre as a so-called ‘offset’ in the model. In addition to this offset, the model included the test centre (*j* =1, ..., 907) and the calendar week (*k* =1, ..., 73) as random intercepts, and the test centre category (pharmacy, doctor’s or dentist’s office, private test centre) as a fixed effect predictor. The calendar week was introduced into the model to account for changes in the positive rates over time, for example induced by changing incidence of COVID-19 infections. Details of the model are described in [6].

The variability between test centres was modeled by centre-specific random intercepts, where each random intercept describes a test centre’s deviation from the general mean. A low centre-specific random intercept indicates a low positive rate for the tests in this centre. Therefore, the reporting of tests by a test centre was considered conspicuous if its estimated random intercept was significantly low (see [6]). For the analysis we used R version 4.2.2 and the R package lme4, version 1.1–31.

#### Deviations from Benford’s Law

**Figure 2:**
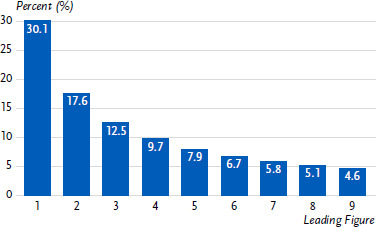
Expected distribution of leading digits according to Benford’s Law. Source: Own figure according to [9]

Benford’s Law [9] (see also [10, 11]) is an observation about the frequency distribution of leading digits in many real-life sets of numerical data. It states that in many naturally occurring collections of numbers, the leading digit is likely to be a small number. Specifically, the probability that the leading digit is 1 is about 30%, while the probability that the leading digit is 9 is only about 5%. Based on Benford’s distribution, the expected probabilities for each number as the first digit are presented in Figure 2.

This law is named after the American physicist Frank Benford, who first described it in 1938. It has been found to hold for a wide variety of datasets, including financial data (e.g. [12]), survey data [13], and physical measurements (e.g. [14, 15]). Researchers have applied Benford’s law to COVID-19 data in several ways, including analysing the distribution of first digits in reported case and death counts (e.g. [16, 17]).

When examining concordance with Benford’s law in the data on claims for COVID-19 tests submitted for reimbursement, we only included test centres with data available for at least 30 days, since the distribution of the first digit is not interpretable otherwise. For each test centre, a chi-squared statistic was calculated. The 10% of test centres with the highest chi-squared statistics were classified as statistically conspicuous. Test centres that claimed reimbursement for less than 30 days were classified as unconspicuous. The analysis was performed with the R package BenfordTests, version 1.2.0; the corresponding analysis code, along with the requirements for applying Benford’s law, can be found in [6].

#### Deviations from the assumption of equally distributed last digits

This method does not examine the first digit, as is the case with Benford’s law, but the distribution of the last digit. The assumption is that the value of the last digit of true scores should be completely random, so that each of the 10 digits (0 to 9) is present with a probability of 10%. Reported test numbers that disproportionately are reported as 0 or 5 have likely been rounded and thus manipulated. Similar to Benford’s law, we only included test centres that had invoiced tests on at least 30 days. The remaining test centres were classified as inconspicuous. The threshold for considering test centres as conspicuous was again set at the 10% with the largest chi-squared statistics value. Requirements for applying the last digit method are described in [6].

### 2.5 Examining the incremental predictive validity of the statistical approaches

To assess the incremental predictive validity of statistical approaches over conventional methods, all statistically conspicuous test centres that had not previously been suspected of fraud by health authorities were subjected to subsequent thorough investigations by the health authorities. Based on data from past and, in some cases, new on-site inspections of conspicuous test centres, a number of factors were considered as possible explanations for outliers in positive rates or daily test volumes, such as opening hours, number of testing booths, number of staff, and location. If possible, potential causes of anomalies in Benford’s law or the last digit approach (e.g., test centres with a constant number of appointments each day) were also investigated. Information from complaints and investigating authorities was used to corroborate or refute the suspicion of fraud. Whether or not fraudulent activities were confirmed in this investigation serves as the dichotomous outcome variable, from which the predictive validity [18] was computed as the share of those for which suspected fraud was corroborated:


(4)

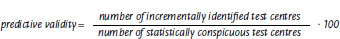



## 3. Results

In the following sections, the results of the statistical inspection of the test numbers invoiced are summarised. A detailed description can be found in [6].

### 3.1 High number of tests per day invoiced

In the first statistical approach aimed at identifying disproportionately high test volumes invoiced, the number of tests invoiced is classified as conspicuous if it falls outside the 90% percentile in terms of the mean number of tests performed per day within a test centre category.

A total of 91 testing centres (6 pharmacies, 39 doctor’s or dentist’s offices, and 46 private testing centres) were classified as conspicuous using this approach. Table 2 summarises these counts in comparison to the conventional approach. The percentage of positive overlap between the conventional and the first statistical method amounts to 24.7% (23/93), the percentage of negative overlap to 91.6% (746/814), and the share of incrementally identified potentially fraudulent test centres identified by the first statistical method which were undetected by conventional approaches amounts to 8.4% (68/814).

### 3.2 Low positive rates identified via Poisson regression

During the reporting period (April 8, 2021 through August 28, 2022), COVID-19 tests were performed on 508 days in 907 test centres; not all centres operated for the entire period. On these days the test centres generated a total of N =118,892 positive rates which were modeled by a Poisson regression. We classified 88 (9.7%) of the 907 test centres as statistically conspicuous with regard to their relatively low rates of positive tests (Table 2). The percentage of positive overlap between the conventional and the statistical Poisson regression approach amounts to 18.3% (17/93), the percentage of negative overlap to 91.3% (743/814), and the share of incrementally identified potentially fraudulent test centres identified by Poisson regression which were undetected by conventional approaches amounts to 8.7% (71/814).

### 3.3 Deviations from Benford’s Law

665 of the 907 test centres had claimed reimbursed for tests on at least 30 days and could thus be included in the analysis of the distribution of the leading digit according to Benford’s Law. Test centres with tests invoiced for less than 30 days were considered unconspicuous. In total, 67 (7,4%) of all 907 test centres were classified as conspicuous. The percentage of positive overlap between the conventional and Benford’s law-based method amounts to 10.8% (10/93) (Table 2), the percentage of negative overlap to 93.0% (757/814), and the share of incrementally identified potentially fraudulent test centres identified by Benford’s law which were undetected by conventional approaches amounts to 7.0% (57/814).

### 3.4 Deviations from the assumption of equally distributed last digits

Testing the assumption of equally distributed last digits was again restricted to 512 test centres with a sufficient number of days for which reimbursement was claimed, the remaining test centres were considered inconspicuous. 52 (5,7%) of all 907 test centres were classified as statistically conspicuous. The percentage of positive overlap between the conventional and the last digit method amounts to 8.6% (8/93), the percentage of negative overlap to 94.6% (770/814), and the share of incrementally identified potentially fraudulent test centres identified by the last digit method which were undetected by conventional approaches amounts to 5.4% (44/814).

### 3.5 Incremental predictive validity of the statistical approaches to identify potential fraud

In the previous sections, we have estimated the incremental contribution of each statistical approach in comparison to conventional approaches to identify potential test centre fraud. Next, we focus on the predictive validity of the statistical approaches. Specifically, among the centres identified as statistically conspicuous, what is the proportion for which the suspicion of fraud could be confirmed after a subsequent thorough examination by the health authorities? Table 3 lists the results, stratified by the four statistical methods used and by different combinations of the methods.

When looking at the methods individually, the high number of tests approach identified the highest number of potentially fraudulent test centres (91 centres). However, the incremental predictive validity (in percentages) over and above the conventional approach was highest for the last digit method (suspected fraud corroborated in 34.6% of the cases), followed by the low positive rate method (30.7%). The absolute number of additionally identified test centres was highest for the low positive rate method, however, since this method could be applied to all test centres, contrary to the examination of the last digit.

Combining methods with the OR operator (statistical conspicuity by at least one method) leads to the highest number (N=49) of test centres with corroborated fraud suspect. However, the proportion of potentially new detected fraudulent test centres was low (21.8%) compared to the performance of the individual methods. With limited testing resources, it is advisable to commence examination with test sites that exhibit statistical anomalies in at least two methods (here, N=61). The health authorities confirmed a high hit rate in these test sites, reaching 41.0%.

## 4. Discussion

The overall aim of this paper was to report on a pilot study comparing the performance of different statistical approaches to identify potential fraudulent test centres in a German city in comparison to conventional fraud detection procedures used by local health authorities by default.

Statistical approaches stand out as more systematic compared to conventional methods, offering distinct advantages in terms of data utilisation, fairness, low error proneness, resource efficiency, and scalability.

Two research questions were empirically addressed: First, what is the level of agreement between conventional methods of identifying suspected test centre fraud and a set of statistical methods applied to data on claims for COVID-19 tests? To address this research question, we have estimated – for each statistical approach – three outcome metrics: The percentage of positive overlap between the conventional and statistical methods compared (paralleling the concept of sensitivity in test accuracy studies), the percentage of negative overlap (paralleling the concept of specificity in test accuracy studies), and the share of incrementally identified potentially fraudulent test centres by the new method, over and above those identified by conventional approaches (corresponding to 1 – negative overlap). The percentage of positive overlap was largest for the high number of tests method, the percentage of negative overlap for the last digit method. The last digit approach, followed by the low positive rate method, also yielded the highest share of incrementally identified potentially fraudulent test centres.

The second research question addressed the predictive validity of the statistical approaches: For a set of test centres identified as conspicuous by the statistical approaches, what is the proportion of those for which a suspicion of fraud could be confirmed after a thorough investigation by the health authorities? Depending on the specific methods and their combinations considered, we have estimated the range of incrementally identified test centres previously undetected by conventional approaches and with confirmed suspicion of fraud between 17.9% and 41.0% of all statistically conspicuous test centres, corroborating the incremental value of the statistical approaches.

Based on these findings, we recommend to make use of all statistical approaches described in this paper in parallel to form a large basis for criminal investigations. If resources are limited, we recommend to investigate those test centres that where classified as conspicuous by at least two of the four methods, which results in the highest incremental predictive validity.

The pilot study presented here suffers from two limitations. First, the preconditions for performing some statistical tests were not always met. For instance, the prerequisite for the application of Benford’s law is that the underlying data have a sufficiently large variance. This assumption is violated for test centres that report similar values every day. Second, the claims data on a daily basis that we had available is of limited use. In an ideal world, we would have had paradata available, allowing us to reconstruct each and every testing procedure, and to assess its trustworthiness. For instance, timestamps would have allowed us to determine whether or not the timeframe within which the test took place was plausible, and whether or not some tests were conducted outside of regular hours of operation. Requiring test centres to use software collecting such paradata and using this data for a continuous monitoring is strongly recommended for future pandemics.

## Key statement

- The study compares statistical methods with traditional fraud detection procedures.
- Some COVID-19 testing centres have fraudulently misrepresented the number of tests conducted to claim higher reimbursements.
- Of the testing centres identified as suspicious by statistical methods, 41% were confirmed as potentially fraudulent through subsequent investigations. These testing centres were previously unsuspected.
- Statistical methods outperformed traditional methods in identifying potentially fraudulent testing centres.
- Mandating the use of software by testing centres that collects detailed test paradata (e.g. timestamps and test results) would further enhance fraud detection.

## Figures and Tables

**Table 1:** Number of test centres, number of test centres suspected of fraud by the health authorities, number of tests and rate of positive tests by test centre category. Source: Data on daily COVID-19 antigen tests from 907 test centres in the period from 8 April 2021 to 28 August 2022

Test centre category	Number of test centres overall	Number of test centres suspected of fraud by the health authorities	Total number of tests	Number of tests per day and test centre			Proportion of positive tests (positive rate in %)
				Mean	Max	Median	
Pharmacy	60	NA	**1,657,633**	108	3,620	64	2.44
Doctor’s or dentist’s office	390	4	**485,434**	21	4,483	5	2.75
Private test centre	457	89	**15,648,440**	194	37,373	115	1.95
**Total**	**907**	**93**	**17,791,507**	**150**	**37,373**	**79**	**2.02**

NA = not available Data on the number of pharmacies suspected of fraud by the health authorities are not available due to pharmacies being under the supervision of another authority.

**Table 2:** Number of test centres (n = 907) by test centre category, suspicion of fraud by the conventional approach, and (not) identified as conspicuous by the four statistical approaches. Source: Data on daily COVID-19 antigen tests from 907 test centres in the period from 8 April 2021 to 28 August 2022

Statistical approach	Test centre category	Suspected of fraud by the health authorities			Not suspected of fraud by the health authorities		
		Statistically conspicuous	Statistically not conspicuous	Total	Statistically conspicuous	Statistically not conspicuous	Total
High number of tests	Pharmacy^*^	0	0	**0**	6	54	**60**
	Doctor’s or dentist’s office	4	0	**4**	35	351	**386**
	Private test centre	19	70	**89**	27	341	**368**
	**Total**	**23**	**70**	**93**	**68**	**746**	**814**
Low positive rate	Pharmacy^*^	0	0	**0**	11	49	**60**
	Doctor’s or dentist’s office	1	3	**4**	15	371	**386**
	Private test centre	16	73	**89**	45	323	**368**
	**Total**	**17**	**76**	**93**	**71**	**743**	**814**
Benford’s Law	Pharmacy^*^	0	0	**0**	5	55	**60**
	Doctor’s or dentist’s office	0	4	**4**	8	378	**386**
	Private test centre	10	79	**89**	44	324	**368**
	**Total**	**10**	**83**	**93**	**57**	**757**	**814**
Last digit method	Pharmacy^*^	0	0	**0**	1	59	**60**
	Doctor’s or dentist’s office	1	3	**4**	14	372	**386**
	Private test centre	7	82	**89**	29	339	**368**
	**Total**	**8**	**85**	**93**	**44**	**770**	**814**

^*^ Data on the number of pharmacies suspected of fraud by the health authorities are not available due to pharmacies being under the supervision of another authority

**Table 3:** Incremental predictive validity of statistical methods in detecting potential billing fraud by testing centres. Source: Data on daily COVID-19 antigen tests from 907 test centres in the period from 8 April 2021 to 28 August 2022

	Statistical approach	Statistically conspicuous test centres [N] (%)			
		Already identified as suspicious by the health authorities via conventional methods	Fraud suspect corroborated through further investigation by the health authorities		Total
			Yes	No	
Statistical methods used individually	High number of tests	23 (25.3)	23 (25.3)	45 (49.5)	**91 (100.0)**
	Low positive rate	17 (19.3)	27 (30.7)	44 (50.0)	**88 (100.0)**
	Benford’s Law	10 (14.9)	12 (17.9)	45 (67.2)	**67 (100.0)**
	Last digit method	8 (15.4)	18 (34.6)	26 (50.0)	**52 (100.0)**
Statistical methods combined	Identified by at least one method	47 (20.9)	49 (21.8)	129 (57.3)	**225 (100.0)**
	Identified by at least two methods	9 (14.8)	25 (41.0)	27 (44.3)	**61 (100.0)**
